# Parasitic Mistletoes of the Genera *Scurrula* and *Viscum*: From Bench to Bedside

**DOI:** 10.3390/molecules21081048

**Published:** 2016-08-17

**Authors:** Ya Chee Lim, Rajan Rajabalaya, Shirley Huan Fang Lee, Kushan U. Tennakoon, Quang-Vuong Le, Adi Idris, Ihsan N. Zulkipli, Natasha Keasberry, Sheba R. David

**Affiliations:** 1 Pengiran Anak Puteri Rashidah Sa’adatul Bolkiah Institute of Health Sciences, Universiti Brunei Darussalam, Jalan Tungku Link, BE 1410 Gadong, Brunei; yachee.lim@ubd.edu.bn (Y.C.L.); rajan.rajabalaya@ubd.edu.bn (R.R.); shirley.lee@ubd.edu.bn (S.H.F.L.); yusri.idris@ubd.edu.bn (A.I.); nazurah.zulkipli@ubd.edu.bn (I.N.Z.); 2 Institute for Biodiversity and Environmental Research (IBER), Universiti Brunei Darussalam, Jalan Tungku Link, BE 1410 Gadong, Brunei; kushan.tennakoon@ubd.edu.bn (K.U.T.); vuong2011732@gmail.com (Q.-V.L.); 3 Biology Faculty, Vinh University, 182 Le Duan, 470000 Vinh City, Nghe An Province, Vietnam; 4 Faculty of Science, Universiti Brunei Darussalam, Jalan Tungku Link, BE 1410 Gadong, Brunei; Natasha.keasberry@ubd.edu.bn

**Keywords:** ethnomedicinal uses, hemiparasite, clinical trials, cancer, antioxidant, *Loranthus*, Santalales, polyphenol, complementary medicine, solid tumor

## Abstract

The mistletoes, stem hemiparasites of Asia and Europe, have been used as medicinal herbs for many years and possess sophisticated systems to obtain nutrients from their host plants. Although knowledge about ethnomedicinal uses of mistletoes is prevalent in Asia, systematic scientific study of these plants is still lacking, unlike its European counterparts. This review aims to evaluate the literature on *Scurrula* and *Viscum* mistletoes. Both mistletoes were found to have anticancer, antimicrobial, antioxidant and antihypertensive properties. Plants from the genus *Scurrula* were found to inhibit cancer growth due to presence of phytoconstituents such as quercetin and fatty acid chains. Similar to plants from the genus *Viscum*, *Scurrula* also possesses TNFα activity to strengthen the immune system to combat cancer. In line with its anticancer activity, both mistletoes are rich in antioxidants that confer protection against cancer as well as neurodegeneration. Extracts from plants of both genera showed evidence of vasodilation and thus, antihypertensive effects. Other therapeutic effects such as weight loss, postpartum and gastrointestinal healing from different plants of the genus *Scurrula* are documented. As the therapeutic effects of plants from *Scurrula* are still in exploration stage, there is currently no known clinical trial on these plants. However, there are few on-going clinical trials for *Viscum album* that demonstrate the functionalities of these mistletoes. Future work required for exploring the benefits of these plants and ways to develop both parasitic plants as a source of pharmacological drug are explained in this article.

## 1. Introduction

Parasitic plants obtain nutrients and water from other flowering plants (hosts) via a well-developed system known as haustoria to connect to the host plants [1,2,3,4]. To date, there are approximately 4500 species of parasitic plants in about 280 genera belonging to 20 families found in all climatic zones except Antarctica and the aquatic environment, including the densely forested Southeast Asia [1]. Apart from a wide range of books and monographs on agricultural pests that encompass only arthropods, nematodes, birds, rodents and other animals that are pests of agricultural importance, there is no review of parasitic plants in Asia.

**Figure 1:**
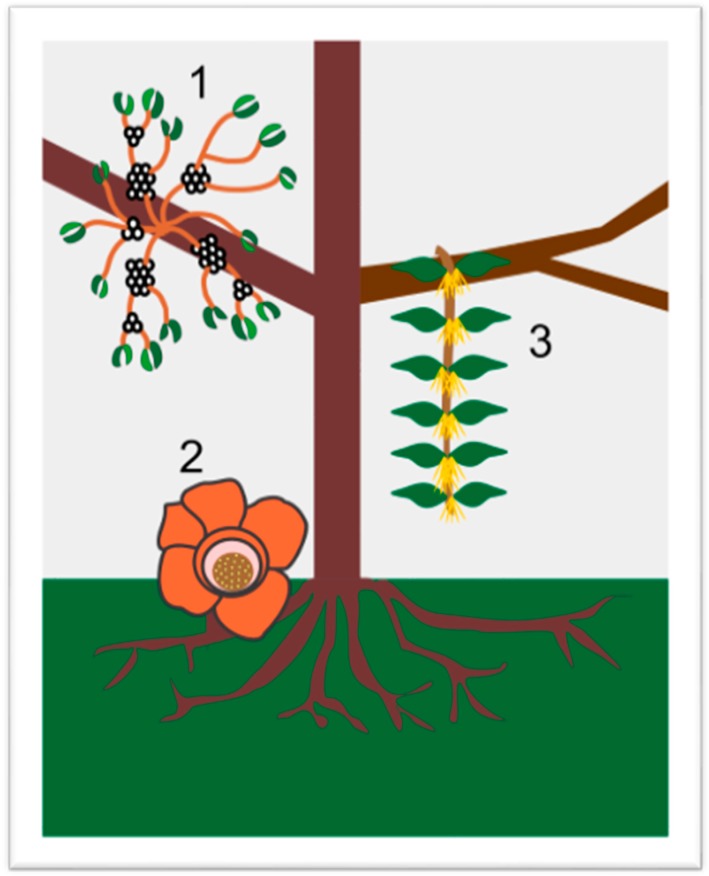
An illustration of aerial parasites. Plant 1 shows *Viscum album*, also known as European Mistletoe. Plant 2 illustrates a root parasite (i.e., *Rafflesia* spp.). Plant 3 depicts *Scurrula ferruginea*, a Southeast Asian mistletoe.

Parasitic angiosperms are classified according to their dependence on hosts for nutrients (hemi- or holoparasitic plants), their required association with hosts (obligate or facultative parasitic plants) or where they attach to hosts (root or shoot parasitic plants). With regard to the carbon dependence of parasitic angiosperms on hosts, most parasitic angiosperms lie between complete heterotrophic xylem- and phloem-feeders and complete autotrophic xylem-feeders [5]. About 4100 species of known parasitic plants are hemiparasites that can get most their carbon needs by own photosynthesis [1]. Approximately 390 species are holoparasites with very low or no photosynthetic capacity, hence they must obtain most or all carbon, water and other nutrients from hosts. There are 40% root parasites [6,7] and 60% aerial parasites, out of which about 1400 hemiparasitic plant species belong to mistletoes [8]. A few common parasitic angiosperms are depicted in Figure 1.

Mistletoes originates from the Celtic name, meaning “all-heal” as they have been used to treat a wide range of human illnesses in the past [9]. Taxonomically, mistletoes belong to one of the three families of the order of Santalales, the Santalaceae (inclusive of the Visacaceae), the Loranthaceae and the Misodendraceae [10]. The two genera that are of interest to us are the *Viscum* genus from the Santalaceae family and the *Scurrula* genus from the Loranthaceae family. Plants from both *Viscum* and *Scurrula* genera have been used as traditional medicinal herbs and documented for their anticancer, antimicrobial, antioxidant and antihypertensive activities. The properties of these plants will be further elaborated in the later sections.

## 2. Parasitic Plant-Host Relationships

Parasitic plant-host relationships have been elucidated by coupling the information of water and nutrient relations from fundamental processes such as transpiration and photosynthesis [5,7]. Parasitic plants generally have negative impacts on associated hosts [5,7,11], which are explained by the competition for resources between parasites and hosts, or the disruption of host photosynthesis induced by parasitism [12,13,14]. Parasitic angiosperms, especially mistletoes and root hemiparasites rarely kill their hosts but modify host physiological function, thus decrease growth, reproduction and competitive ability of infected hosts under most circumstances [7,15,16,17]. In contrast, some studies reported the positive effect of parasitic plants on performance of infected host plants. For example, by the holoparasitic stem parasite *Cuscuta reflexa* increased stomatal opening, transpiration rate and net photosynthesis of infected *Coleus blumei* and *Ricinus communis* host plants due to a sink-source effect but still suppressed the growth and dry matter accumulation in hosts [18,19]. Increase in net photosynthesis of host leaves due to parasitic-host plants’ sink-source effects were also reported in a number of other studies [20,21]. The enhanced effects on photosynthesis of infected hosts seems only being induced by holoparasite parasitism rather than hemiparasites [22].

Host nature might influence the growth of parasites and Table 1 illustrates a few parasitic plants with their commonly known host plants. Although how the host affects performance of parasites is less studied [7], nevertheless, it was reported that the increased resource uptake by the host *Pinus ponderosa* increased resources to the mistletoe *Arceuthobium vaginatum* subsp. *cryptopodum* [23]. Similarly, photosynthetic parameters, foliar traits and mineral accumulations of the mistletoe *Dendrophthoe curvata* parasitizing different host species varied significantly [24,25,26]. The mistletoe *Viscum album* also exhibited different levels of total phenolic acids and antioxidant activity when parasitizing different host species [24]. Thus the effects of hosts on performance of parasites really exist, however, further elucidation of parasitic plant response to host condition is needed [7]. Parasitic angiosperms can provide a valuable feedstock for pharmaceutical industry, therefore their medicinal values should be considered in taking account the nature of host species they are parasitizing as well as regional specificity.

In this review, we will focus on parasitic plants of the genera *Scurrula* and *Viscum*. The former is a prevalent genus of parasitic plants found in Asia with little information in the literature about any clinical trials, while the latter, the European mistletoe *Viscum album* has undergone many clinical trials in Germany and other European countries. Table 2 lists a few examples of the *Scurrula* plants with their other known names. Evidence of the ethnopharmacological effects of *Scurrula* parasitic plants has been sparsely documented. Unravelling the knowledge of phytochemical and ethnopharmacological effects will open up avenues for drug design and treatment of various ailments. Analysis of literature review on the clinical trials of *Viscum album* will shed light on similar potential of *Scurrula* plants. Nevertheless, the potential of *Scurrula* plants is not limited to the knowledge gained from *Viscum album*.

## 3. Phytochemistry

The *Scurrula* species has not been extensively studied in terms of its phytochemistry. Although a few studies have identified flavonols to be present in the *Scurrula* species [38,39,40], only one paper has isolated and elucidated the structures of flavonols from *Scurrula ferruginea*, and subsequently reported their cytotoxicity on human cancer cell lines (DU145, K562, MCF-7, U251) [41]. Lohézic-Le Dévéhat et al. isolated three flavonols: quercetin, quercitrin, and 4″-*O*-acetylquercitrin, the latter acetylated derivative is uncommon in higher plants. The acetylated quercitrin (2″-, 3″- and 4″-) had previously been isolated in several flowers and ferns, but not in the *Scurrula* genus. Quercitrin has recently been found to mediate the protective effects on endothelial progenitor cells, that in turn, repair damage endothelium and have anti-atherosclerosis effects [42]. Quercetin exerts anti-proliferative effects on cancer cells by growth arrest of HepG2 cells [43], as well as targeting microRNA-21 signalling in BEAS-2B cells [44]. Therefore, both quercetin and quercitrin have potential therapeutic effects.

Moghadamtousi et al. have also isolated another family of chemicals known as coriaria lactones from the *Loranthus parasiticus* [45]. *Loranthus parasiticus* is a hemiparasitic plant and as such it derives its phytochemical constituents and subsequently the biological activity from its respective hosts. The coriaria lactones include sesquiterpinoids such as coriamytrin, tutin, corianin, and coriatin. These compounds are not usually native to the *Scurrula* species, and it was indicated that these compounds were directly transported from the host plant to the mistletoes [45]. Table 3 lists the chemical structures of compounds found in *Scurrula* plants.

Despite the limited phytochemistry research on *Scurrula*, there are reports of the isolation of various chemicals from *Scurrula* plants. Flavonoids in general have been studied to determine the relationship between structure and biological activity [49]. Flavonoids have been shown to act as inhibitors of various enzymes in cellular processes, such as protein kinase and topoisomerase [49]. Deregulation of these enzymes has been closely linked to various diseases [50,51]. Therefore, inhibition of these enzymes would be a useful strategy towards cancer therapy and treatment of other related diseases.

## 4. Ethnomedicinal Uses

### 4.1. Anti-Cancer Effects

Various mistletoe species have been traditionally used to treat cancer in Indonesia, including *Scurrula ferruginea* and *Scurrula oortiana* (Indonesian tea mistletoe). In a screen for antiviral and cytotoxic activities of various Indonesian medicinal plants, *Scurrula ferruginea* exhibited significant cytotoxic activity, particularly against U251 glioma cells, with an IC_50_ (50% inhibitory concentration) of 19 µg/mL [52]. Further investigations on *Scurrula ferruginea* was followed by isolation of the flavanol quercetin and two other flavanols from the plant. Quercetin proved to be the compound responsible for the cytotoxic activity on glioblastoma cells, with an IC_50_ of 35 µM [41].

However, other *Scurrula* species have been the focus of various scientific studies looking at the anti-cancer properties of the mistletoe plants. Ohashi et al. used a bioassay guided separation of compounds to isolate various bioactive compounds from *Scurrula atropurpurea*. They have identified octadeca-8,10,12-triynoic acid as the main biologically active compound in the anti-cancer activity of *Scurrula atropurpurea* using the MM1 cell invasion assay. Octadeca-8,10,12-triynoic acid is able to inhibit most of the invasion of the tumour cells in rats. C18-triyne fatty acid and the C16-triyne fatty acids were also identified as potent cancer cell-invasion inhibitory compounds in a second study by the same group. They concluded that the number of unsaturated bonds in the fatty acids appear to strengthen the invasion inhibition activity of a compound [46,47].

A study investigating effect of the extract of *Scurrula atropurpurea* found that the treatment of HeLa cells with plant extract led to decreased expression of murine double minute 2 (MDM2), which acts as an inhibitor of the p53 tumour suppressor, which, in turn, is an activator of p21 [53,54]. Conversely, p21 levels were found to be increased in this study. Additionally, Bax protein expression was also significantly increased, suggesting the activation of apoptosis via the intrinsic pathway [53].

TNFα is a cytokine produced by activated macrophages that has potent anti-cancer activities [55], and has potential as an anti-cancer treatment [56,57]. Murwani et al. studied extracts of *Scurrula oortiana* to investigate whether the response of tumour cells towards TNFα is affected by the extracts. The assay used consisted of measuring changes in cell viability in TNFα-mediated lysis, after treatment with plant extracts. The author found that the stem extract was more effective in modulating the response of WEHI-164 cancer cells towards TNFα treatment, and made the cells more sensitive to cell lysis due to TNFα [31]. Nevertheless, administration of TNFα has been associated with systemic toxicity [58,59]. A recent study found that engineered TNFα-expressing tumor cells target towards malignant cells and release TNFα locally to shrink the tumours. In addition reducing side effects such as systemic toxicity mentioned, this targeted tumour delivery circumvents acute inflammation and weight loss as well. Therefore, the use of TNF-expressing tumor cells for drug delivery shows potential [60].

Similar to *Scurrula* extracts, *Viscum* extracts also possess TNFα activity. Aqueous extract of Korean mistletoe (*Viscum album coloratum*) retards tumour metastasis via expression of TNFα [61]. The production of TNFα, as well as other cytokines, is due to increase in the quantity and cytotoxic effect of natural killer cells [62]. This increase of the natural killer cells illustrates the strengthening of the immune system to inhibit cancer growth without causing side effects. It must be borne in mind that TNF-α is documented to play a double-edged role in tumors: while higher levels are anti-tumoural, lower levels induce cancer, angiogenesis and metastasis [63]. Therefore the use of extracts from mistletoes, either *Scurrula* or *Viscum*, have shown anticancer and immunomodulatory effects that enhances its role as anticancer agent. These mistletoes also possess antioxidative and other beneficial effects, such as antimicrobial and antihypertensive effects as explained in later sections.

Pentacyclic triterpenes present in *Viscum album* lipophilic extract are known for their immunomodulatory and anticancer properties through macrophages. Data from the study demonstrates their positive influence on modulation of in vitro monocyte chemotactic transmigration resulting in anticancer activity. This also showed down regulation of IL-6 and up-regulation of TNF-α. Moreover, the site of occurrence of tumors influences the role of TNF-α as pro- or anti-tumour agent [63]. Tumor cells suppress the dendritic cells due to the secretion of IL-10 and TGF, however *Viscum* preparations stimulate the maturation and activation of human dendritic cells, which may facilitate anti-tumoral immune responses [64], thus substantiating their role as complimentary therapy in cancer.

PGE2 overproduction is observed in many pro-tumoral conditions in association with COX-2, leading to inflammation. As known through various studies there is an intricate relationship between inflammation and cancer. Many anti-tumour phytotherapeutics are pronounced anti-inflammatory agents. *Viscum* preparations exhibited dose-dependent reduction of PGE2 secretion in A549 cells which was proportional to reduced selective COX-2 expression, which was beneficial in reducing the side effects [65]. Moreover, it was demonstrated by the same group of researchers that the mechanism of down regulation of COX-2 was by the destabilization of COX-2 mRNA [66] in the human lung adenocarcinoma cells.

*Viscum album* lectins stimulated proliferation of CD4+ T-cells which attributes to the anti tumour activity [67]. Mistletoe triterpene acid solubilized in cyclodextrin was investigated for its’ effect on C.B-17/SCID model of pre-B Acute lymphoblastic leukaemia (NALM-6). The results indicated a dose-dependent apoptosis induction via pathways dependent on caspase-8 and -9, which prolonged mean survival [68]. Subsequently, mistletoe triterpenes solubilized in cyclodextrins were investigated along with solubilised triterpene acids or *Viscum* lectins for cell proliferation on human acute myeloid leukaemia cells. Dose-dependent induced apoptosis was observed through the caspase-8 and -9 dependent pathways. Simultaneous down-regulation of apoptosis inhibitor members and proteins of Bcl-2 family was elucidated from the experiments. Moreover, synergism of all three extracts and significant reduction of tumour weight was demonstrated in acute myeloid leukaemia mouse model experiments; the therapeutic effectiveness of *Viscum* triterpenes was comparable to cytarabine [69].

Smoldering inflammation is of interest in cancer as it is has been proposed as cancer’s seventh hallmark. The innate leukocyte macrophages release reactive nitrogen and oxygen intermediates inducing DNA damage; also these cells promote chronic inflammation state by the secretion of (TNF)-α, interleukin (IL)-6 and IL-1β, proinflammatory cytokines [70].

The study of *Scurrula* is still in its infancy and so far, there are no documented clinical trials on *Scurrula*. On the other hand, *Viscum album* has been developed into drugs, with the most well-known one named Iscador. Currently, Iscador is the most commonly used oncological drug in Germany. In other parts of Europe, Iscador is used as complementary cancer therapy. Iscador, *Viscum album* extract, has been found to prolong survival of cancer patients and seems to stimulate self-regulation [71]. As a complementary treatment, Iscador has shown to improve cancer-related fatigue without causing toxicity [72].

*Viscum album* extracts have mostly been used as adjuvant therapy in cancer patients [73]. However, individual case studies suggest that there are may be direct benefits to using *Viscum album* extracts as the main therapy in cancer patients [74,75]. *Viscum album* extracts exert their anti-cancer effects both by modulating the immune system as well as directly having cytotoxic activities on cancer cells, with a less potent effect on normal, healthy cells [73,76]. Remarkably, fermented *Viscum album* extracts are additionally able to reduce the metastatic potential of glioblastoma cells in vitro [76]. The migratory activity of 3T3 mouse fibroblasts and HaCat keratinocytes are also reduced, suggesting that this is not a cancer cell-specific effect [77]. These data point to the potential of mistletoes as main drugs as well as usage in supportive care.

Mistletoes, like *Taxillus chinensis*, have been found to inhibit fatty acid synthase (FAS) [78]. FAS is produced in low levels in most tissues, except for the liver, adipose tissue, and lactating mammary gland. In carcinomas, however, FAS expression is upregulated [79]. Therefore, FAS is a potential anti-cancer therapy. Cerulenin, C75 and orlistat are early small-molecule FAS inhibitors that induce apoptosis or delay tumour growth in several cancer cell lines and cancer xenograft models, respectively. However, their mechanism of action remains unknown. Due to their side effect such as stimulation of weight loss, they are not being pursued for development into systemic drugs [80].

A recent study has shown that a novel FAS inhibitor TVB-3166, induces apoptosis in breast and prostate cancer cell lines, without affecting non-cancer cell lines. In addition, weight loss was not significant in TVB-3166 treated mice compared to vehicle-treated mice. TVB-3166 alters lipid raft distribution in the membrane, disrupts palmitoylated protein localization, and inhibits the PI3K-AKT-mTOR and β-catenin signal transduction pathways as well as modulate the expression genes involved in metabolic, proliferative and apoptotic genes [97]. The authors postulated that TVB-3166 could provide a novel anti-cancer therapy due to their selectivity, potency, reversible mode of action and in vivo availability [97].

Dietary products rich in phytoconstituents which may inhibit FAS are potential natural alternatives for anti-cancer therapy [80]. Screening phytoconstituents in mistletoes, especially in the underexplored *Scurrula ferruginea* for bioactive FAS inhibitors with minimal toxicity/side effects and understanding their mode of action and associated target signaling pathways would pave the way for the identification of novel, anti-cancer biomolecules.

Investigations of the anti-cancer activities of various mistletoe species are few, but promising. Interestingly, various compounds isolated from different mistletoe species appear to exert their cytotoxic activities on cancer cells through different mechanisms. Therefore, a comprehensive study on the anti-cancer activities of the various mistletoe species would prove useful for anti-cancer therapy. Table 4 summarises the biological activities of compounds isolated from parasitic plants.

### 4.2. Anti-Microbial Effects

Indonesian plants have been traditionally used by the indigenous people for generations as their main source of medicine against infections. Several *Scurrula* plants members have been shown to possess promising anti-microbial activity. A study by Marvibaigi et al. [82], showed that the leaf, flower and stem *Scurrula ferruginea* crude acetone extract had good anti-bacterial activities against a range of Gram positive and negative bacteria. Notably, the leaves and stems *Scurrula ferruginea* extract has potent anti-microbial activity against the clinically relevant skin pathogen, *Staphyloccocous aureus* [82]. This observation corroborates previous findings with crude methanol extracts from another closely-related *Loranthus* family member *Loranthus micranthus* species, where potent anti-bacterial activity was observed against *Staphyloccocous aureus* and other bacteria including *Bacillus subtilis* and *Pseudomonas aeruginosa* [84]. Whole *Scurrula atropurpurea* plant extract was shown to be inhibitory using an agar well diffusion assay technique against a number of Gram negative bacteria including *Bacillus subtilis*, *Klebsella pneumoniae*, *Vibrio cholerae* and *Escherichia coli* [83]. Leaf and stem crude ethanol extracts from *Scurrula atropurpurea* have been shown to have growth inhibiting activity against *Enterobacter sakazakii* in an agar disc diffusion assay [81]. Commonly found in infant formula milk, *Enterobacter sakazakii* accounts for over 50% of premature infant death from sepsis and meningitis [98]. In contrast, no killing activity was seen with the same leaf extract on bacteria isolated from a skin wound from a mouse skin wound healing model [99]. However, the study excluded several important experimental details in the way their animal model was established and the procedure detailing their anti-microbial assay was incomplete, therefore questioning the veracity of their data. Nonetheless, majority of the evidence show that *Scurrula* plants have an intrinsic anti-microbial ability against a broad spectrum of different types of bacteria. However, current work only focuses on the anti-bacterial effect of crude extracts. Future work looking at the anti-bacterial effects of further fractionated extracts is needed to determine the most antibacterial active fraction for further downstream drug discovery applications. These findings could open the possibility to potential chemical classes of antibiotics and selective agents for infectious diseases management and control.

Ethnopharmacological studies show that indigenous people of Indonesia could diagnose viral infections and could cure them by using specific Indonesian-native plant preparations [100]. Although the evidence is limited, extracts from *Scurrula* plants have also shown to possess anti-viral properties. Crude methanol *Scurrula ferruginea* extracts inhibited replication of poliovirus with a remarkable IC_50_ of 62 µg/mL [41]. In contrast, the same extract had no observable inhibitory activity against herpes simplex virus-1 (HSV-1) [52]. *In ovo* injection of crude *Scurrula oortiana* extract preparations into embryonated chicken eggs was shown to be protective when challenged with Marek’s Disease virus (MDV), a highly contagious virus that causes poultry disease [32].

The potential anti-viral effects of extracts *Scurrula* plants against other viruses have not yet been investigated. Given the strong ethnopharmacological evidence it is possible that *Scurrula* plants can be effective against a range of viruses, at least to those viruses that are endemic to the people in a particular region.

A handful of studies have also investigated the anti-microbial effects of *Viscum* plants. The first demonstration of this was the clear inhibitory effect of some *Viscum album* subspecies (ssp.) extracts on the growth of *Mycobacterium tuberculosis*, a pathogen that causes tuberculosis [101]. In a microplate Almar blue assay (MABA), an anti-tuberculous assay, ethanol extracts from ssp. *album*, ssp. *austriacum*, ssp. *abietis* and ssp. *austriacum* exhibited anti-mycobacterial activity at a minimum inhibitory concentration (MIC) of 200 µg/mL. Another study showed that extracts from *Viscum album* ssp. *abietis* showed varying anti-microbial effects on a range of bacteria and fungi [102]. The disc diffusion assay showed that different fractions of *n*-hexane extract of *Viscum album* ssp. *abietis* varying levels of anti-bacterial activities against *Bacillus subtilis*, *Staphylococcus aureus*, *Escherichia coli*, *Pseudomonas aeruginosa*, *Enterobacter cloacae* and *Proteus vulgaris* and anti-fungal activity against *Candida albicans*. Using a similar assay, another study similarly showed anti-bacterial activity of ethanol extracts of *Viscum album* leaves on common bacterial pathogens *Staphylococcus aureus*, *Escherichia coli* and *Proteus vulgaris* [103]. Later work by Hussain et al. 2011 [104] showed that a range of different leaf and twig extracts of *Viscum album* showed prominent activity against several bacteria, including *Staphylococcus aureus*, *Bacillus subtilis*, *Escherichia coli*, *Bordetella bronchisiptica*, *Pseudomonas aeruginosa*, and *Pseudomonas syringae*. Overall, the evidence to date suggest that extracts from *Viscum* plants may possess promising antibacterial properties. However, the active antimicrobial constituent responsible for this effect and its subsequent mode of action remains to be investigated. In addition to the actual *Viscum* plant, work should also focus on the potential antibacterial properties of the endophytic fungi that lives on the plant. Recent work by showed that lectin isolated from endophytes from *Viscum album* had good inhibitory activity on the growth of a number of tested pathogenic bacteria including *Proteus mirabilis, Serratia marcescens* and *Staphylococcus aureus* [105]. Interestingly, *Viscum* plants may possess potential anti-viral activity. In a plaque formation assay on human parainfluenza virus type 2 in Vero cells, the aqueous leaf extract of *Viscum album*. ssp. *album* was found to reduce plaque numbers [106]. When the differential sensitivity of HPIV-2 to the aqueous extract was further studied by virus yield reduction assay in Vero cells, the results showed that production of the infectious virus decreased over 72 h suggesting that the aqueous extract interferes with a subsequent stage of the virus replication cycle within the cell. However, further work with other viruses is needed to assess the spectrum of the anti-viral activity of *Viscum* plants extracts.

### 4.3. Antioxidant Properties

Both reactive nitrogen species (RNS) and reactive oxygen species (ROS) are common forms of free radicals. Examples of ROS are hydroxyl radical (OH•), hydrogen peroxide (H_2_O_2_), superoxide anion (O_2_^−^•) as well as the peroxyl radical (HO_2_•), whereas nitrogen dioxide radical (NO_2_•) radical and nitric oxide radical (NO•) are some examples of RNS. Any atom or molecule with one or more unpaired electrons is a free radical [107].

Free radicals can be generated within cells, as normal by-products of aerobic metabolism (mitochondrial respiration) [108]. Free radicals can also be derived from external sources including alcohol, smoking, medication, pollution and radiation [108,109]. Excess ROS are capable of membrane damage, changing the interior shape of proteins to alter their structure and function, denaturation of lipids, inducing structural damage to DNA and apoptosis [110]. Accumulation of excess ROS in the body may cause oxidative stress, which is thought to contribute to the development of various diseases such as ageing, autoimmune disorders, cancer, cataract, rheumatoid arthritis, neurodegenerative diseases and cardiovascular diseases. Therefore, the human body has evolved mechanisms to detoxify ROS-induced oxidative stress by generating antioxidants which are scavengers of ROS [111]. As endogenous antioxidant defenses are insufficient to prevent free radical-induced damage completely, diet-derived antioxidants (vitamins E and C, carotenoids and plant pigments) are therefore crucial to combat ROS-induced oxidative damage for optimal health [109,112].

Plants are enriched in beneficial bioactive compounds with antioxidant properties. Majority of antioxidant compounds present in plants are secondary metabolites-phenolic compounds (tannins, flavonoids and phenolic acids) which exhibit their protective properties through scavenging free radicals [113,114,115]. In addition to phenolics, plants may also contain antioxidant volatile oils, carotenoids and vitamins [82].

In a recent study, the total phenolic content of stem, leaf and flower extracts of *Scurrula ferruginea* were assessed and the data suggested that the stem extract contained the highest level of phenolic compounds [82]. Furthermore, the phenolic content of the respective plant parts correlates to their antioxidant properties: the highest antioxidant activity was found in the stem, followed by the leaf and flower extracts of *Scurrula ferruginea* [82]. Interestingly it was also shown that the mature leaf extract of *Scurrula ferruginea* exhibited higher antioxidant activity than dried tender leaves as indicated by four different in vitro antioxidant assays: ascorbate iron (II) induced lipid peroxidation inhibition assay, DPPH (2,2-diphenyl-1-picrylhydrazyl) free radical scavenging activity, ferric reducing antioxidant potential and total phenol content [90]. This is concordant with the findings that harvesting period or seasonal factors may account for differences in phytoconstituents [116] and hence bioactivity between tender and mature leaf extracts. In addition, the methanolic leaf extract of *Scurrula ferruginea* showed higher antioxidant activity in comparison to the aqueous leaf extract [82].

Although there is only one paper published (to date) supporting the antioxidant role of *Scurrula* ferruginea [82], within the Loranthaceae plant family, various related species of the *Loranthus* genus have been widely investigated for their antioxidant activity. The leaves and leafy twigs of the African mistletoe, *Loranthus micranthus* are enriched with polyphenols. Polyphenols and phenolic glycosides isolated from methanol extracts of *Loranthus micranthus* leafy twigs parasitic on the host *Hevea brasiliensis* demonstrated significant antioxidant activity through a 2,2-diphenyl-1-picryl-hydrazyl (DPPH) radical scavenging assay. Among the tested polyphenols, tutin and peltatoside in particular exhibited the most prominent radical scavenging activity [117]. Meanwhile, from the methanol extract of *Loranthus micranthus* leaves parasitic on host *Kola acuminata*, −(−) catechin-7-*O*-rhamnoside, −(−) catechin-3-*O*-rhamnoside and 4′-methoxy-catechin-7-*O*-rhamnoside are the most potent antioxidant polyphenols [118].

The antioxidant properties of the crude (methanol) extract obtained from the aerial parts (leaves, flowers and twigs) of another species, *Loranthus regularis* were also tested. Partitioning of the crude (methanol) extract results in subfractions of petroleum ether, chloroform, ethyl acetate extract and *n*-butanol fractions. As the crude extract and its associated ethyl acetate fraction demonstrated the highest antioxidant activity, it was postulated that majority of the antioxidant compounds are enriched in the ethyl acetate fraction. Indeed, activity-guided fractionation and repeated column chromatography of the ethyl acetate fraction, in combination with mass spectrometry and comparing the spectral data to known literature compounds lead to isolation/identification of three novel quercetin glycosides in *L. regularis*: quercetin 3-*O*-β-d-galactopyranoside; (2) quercetin 3-*O*-β-l-arabinopyranoside and (3) quercetin 3-*O*-α-l-rhamnopyranoside [89]. These quercetin glycosides have been previously identified in *Loranthus tanakae* [119]. At 50 and 100 μg/mL, the crude extract, ethyl acetate and *n*-butanol fractions as well as the isolated quercetin glycosides of *Loranthus regularis* exhibited considerable antioxidant activity as demonstrated through their ability to scavenge DPPH free radical where the antioxidant activity was similar to that of ascorbic acid (positive control). Meanwhile, only the ethyl acetate and *n*-butanol fractions (at 1 mg/mL), and the isolated quercetin glycosides of *Loranthus regularis* demonstrated antioxidant properties through the alternative β-carotene/linoleic acid antioxidant assay [89].

Increased oxidative stress in the brain and decreased antioxidant capability is a key factor in the etiology of neuropsychiatric diseases [110]. As the brain is a major centre for oxygen metabolism, it is especially vulnerable to ROS-induced oxidative stress [110]. As various independent groups have demonstrated the neuroprotective role of antioxidants [120,121,122,123], the neuroprotective activity of phenol-rich/antioxidant potent *Loranthus parasiticus* against hydrogen peroxide (H_2_O_2_)-induced oxidative damage in NG108-15 hybridoma (mouse neuroblastoma x rat glioma hybridoma cells) were also investigated. Neuroprotection was assessed by performing a MTT (3-(4,5-dimethylthiazol-2-yl)-2,5-diphenyltetrazolium bromide) assay, a cell viability assay. MTT assay results indicate that the aqueous fraction of *Loranthus parasiticus* (LPAF) is highly neuroprotective [92]. The neuroprotection is postulated to be attributed to two different molecules (proanthocyanidins) isolated from the LPAF: (+)-catechin and AC trimer, with (+)-catechin demonstrating more potent neuroprotective activity. The proanthocyanidin (+)-catechin improved cell viability and decreased the levels of intracellular ROS in a dose-dependent manner against H_2_O_2_ induced oxidative stress in NG108-15 hybridoma cells. (+)-catechin also prevents the termination of mitochondrial membrane potential (MMP), and inhibited cell cycle arrest at sub-G1 population following H_2_O_2_ insult. Besides decreasing ROS generation, (+)-catechin also decreases the early and late apoptotic population (as indicated by annexin V staining, which binds to externalized phosphatidylserine, upon apoptotic cell death). (+)-catechin is a flavanol abundant in green tea well-characterized for its anti-cancer and neuroprotective roles [124]. Therefore, the presence of proanthocyanidins in this parasitic plant has therapeutic potential for treatment of oxidative stress-induced neurological disorders such as Alzheimer’s and Parkinson’s disease.

In South-West China, *Loranthus parasiticus* has been used for the treatment of schizophrenia [92]. Ethanol extracts of *Loranthus parasiticus* leaves contains *s*esquiterpene lactones such as corianin, coriamyrtin, coriatin and tutin which are the postulated anti-schizophrenic compounds [45]. Studies have also shown that the aqueous fraction of *Loranthus parasiticus* exhibit high antioxidant activity (in a dose-dependent manner), compared to the methanol and ethyl acetate extracts. Concordantly, the aqueous fraction of *Loranthus parasiticus* contains the highest total phenolic content comprising anthocyanins, flavonoids, phenolic acids, and tannins [45]. Furthermore pre-treatment with LPAF can restore the intracellular antioxidant glutathione (GSH) level in a concentration-dependent manner after NG108-15 cells were treated with H_2_O_2_ [91]. These data suggest and support the potent role of antioxidants in neuropsychiatric diseases by restoring intracellular antioxidant levels as well as removing free radicals.

As free radicals may induce initiation of cancer through DNA damage and mutations [125], it can be postulated that scavenging free radicals with antioxidants is anti-cancerous (cytotoxic). Interestingly, studies have also demonstrated the neuroprotective (anti-cytotoxic) role of antioxidants. The dual role of antioxidants, cytotoxic or neuroprotective, can be explained by the cell-type specific role of antioxidants or synergistic effects between different antioxidant molecules. Nevertheless, all these studies show that antioxidant therapy is an attractive option for both cancer and neurodegenerative diseases among others.

The semi-parasitic nature of *Loranthaceae* family members may account for differences in composition as well as amount/activity of bioactive compounds isolated from each genus or species within a genus. Host-parasite-specific interaction may modulate generation and therefore metabolism of bioactive compounds within the *Loranthaceae* family, resulting in differences in availability/composition of phytoconstituents. Geographic differences may also account for these differences between species of the genus *Loranthus*. Indeed, it has been shown that seasonal factors (harvesting time) may account for differences in constituent composition/components of *Loranthus micranthus* [116].

While the antioxidant effect of *Scurrula ferruginea* remains under-reported, there are several studies documenting the antioxidant properties of its European counterpart, *Viscum album* (European mistletoe). Pre-treatment of HeLa cells with methanolic extracts of *Viscum album* parasitic on three host trees (locust tree, lime tree, and hedge maple tree) inhibits oxidative mitochondrial DNA damage and the effect of this inhibition is dependent on the host tree. The extract from *Viscum album* parasitic on lime tree and locust tree completely suppressed oxidative mitochondrial DNA damage, whereas that from the hedge maple tree is only half as effective in inhibiting oxidative mitochondrial DNA damage [94].

A recent study also proposed the use of *Viscum album* ethanolic extracts (semiparasitic on the host tree *Quercus* *acutissima*) as a natural source of antioxidants/preservatives in uncooked meat products. This is due to various antioxidant assays (free radical scavenging activity, superoxide anion radical scavenging and hydroxyl radical scavenging activity) supporting the antioxidant potential of *Viscum album* extracts due to their high phenolic and flavonoid contents [93].

Another study utilizing HPLC to analyze the phenolic compounds composition of *Viscum album* leaf and stem extracts from northwest Romania parasitic on five different host trees, *Acer campestre* (VAA), *Mallus domestica* (VAM), *Fraxinus excelsior* (VAF), *Populus nigra* (VAP) and *Robinia pseudoacacia* (VAR) identified 17 compounds and these include a pentacyclic triterpene (betulinic acid), 12 phenolic acids (gallic acid, protocatechuic acid, gentisic acid, chlorogenic acid, *p*-OH benzoic acid, caffeic acid, syringic acid, salicilyc acid, *p*-coumaric acid, ferulic acid, sinapic acid, and *trans-*cinnamic acid) and four polyphenols (naringenin, quercetin, kaempherol and rosmarinic acid) [126]. In addition, quantitative HPLC analysis also showed that the leaves of *Viscum album* contain higher concentration of bioactive compounds compared to stems [126].

Similar to *Scurrula ferruginea*, the phytoconstituents of *Viscum album* is affected by seasonal factors (harvesting time) and the nature of the host tree [127]. This may therefore explain the varying phenolic contents and hence antioxidant potential of *Viscum album* leaves and stem extracts harvested at different seasons and also from different host trees. It has also been reported that younger plants (harvested in May) have higher antioxidant potential compared with older plants (harvested in July the same year) [128].

Unlike its European counterpart, *Scurrula ferruginea* stem extracts elicit higher antioxidant activity (due to their higher phenolic content) compared to the leaf extract, whereas *Viscum album* leaf extracts are postulated to have higher antioxidant potential compared to the stem extract due to lower concentration of bioactive compounds present in the stem extract. Although, *Scurrula ferruginea* flower extract demonstrates some protection against oxidative damage [82], the antioxidant potential of *Viscum album* flower extract remains to be investigated. The other major difference between *Scurrula ferruginea* and *Viscum album* in terms of antioxidant capability is the maturity level of leaves; *Scurrula ferruginea* mature leaf extracts tend to have higher antioxidant capabilities compared to tender leaf extracts [90] whereas in the case of *Viscum album*, young leaf extracts have more potent antioxidant effects [128].

Comparing the phytochemicals present in *Viscum album* and *Scurrula ferruginea* and understanding how various factors affect differences in composition of constituents within all mistletoe families are also valuable avenues to explore. In addition, understanding how host-parasite interaction, geographical and seasonal variation, harvesting time and, maturity of leaves affects phytoconstituents in mistletoes *Viscum album* and *Scurrula ferruginea* will provide useful insights in terms of optimizing isolation of antioxidant rich bioactive compounds. As there is only one study published to date regarding the antioxidant role of *Scurrula ferruginea*, further studies that need to be addressed in the future include profiling of phytoconstituents present in *Scurrula ferruginea* and other genus of the *Loranthaceae* family, as well as dissection of the role/biosynthetic pathway of all bioactive plant compounds with antioxidant properties. Identification of bioactive plant constituents may help maintain antioxidant status and confer protection against free radical damage-induced diseases including cancer and neurodegeneration.

### 4.4. Antihypertensive Effects

By 2030, approximately 23 million people are projected to die per annum from cardiovascular complications. Hypertension, an important risk factor for cardiovascular disease, has been found to be modulated by the methanolic extract of *Scurrula ferruginea*. The vasorelaxant properties of *Scurrula ferruginea* using both in vitro and in vivo animal experimental approaches were studied. The aerial parts of the plants (leaves, fruits and flowers) were collected, dried and pulverized into powder. Hot extraction of the powder with chloroform, petroleum ether, methanol, ethyl acetate and water was then carried out, generating five different extracts. Various responses of isolated rat aortic ring towards different doses of noradrenaline in the presence of the extracts were measured. The methanolic extract produced a significant dose-dependent inhibition in the maximum response, suggestive of containing non-competitive inhibitory activity compounds. This result is corroborated by the in vivo experiments where the most potent extract for blood pressure lowering activity in anesthesized normotensive Sprague Dawley rat model was the methanolic extract [86]. This study is limited to the effect of the vasoconstriction or dilation with respect to noradrenaline in the presence of the extract and suggests that the management of hypertension is via vasodilation of isolated rat aortic vessels that may not reflect in vivo situations.

To investigate the hypotensive mechanism of the extract, the methanolic extract of *Scurrula ferruginea* was obtained using Soxhlet extraction method. These extracts were administered into Sprague Dawley (SD) rats in intravenous boluses of 25, 50, 100 and 200 mg/kg concentrations. The results illustrate that there was a dose-dependent reduction of the mean arterial pressure of the rats. This hypotensive effect of the extract was reduced in the presence of atropine, a nonselective muscarinic antagonist and L-NAME (Nω-nitro-l-arginine methyl ester), a nitric oxide synthase (NOS) inhibitor. This dose-dependent effect of the methanolic extract of *Scurrula ferruginea*, together with the reduction of effect in the presence of atropine, was replicated in guinea pig ileum. Conversely, neostigmine (acetylcholinesterase inhibitor) enhanced the contraction of the guinea pig ileum. These observations led to a conclusion that the methanolic extract of *Scurrula ferruginea* exerts is hypotensive effect via stimulation of muscarinic receptors and/or stimulation of nitric oxide (NO) release [86]. The work by Ameer to pinpoint the mechanism the hypotensive effect of the methanolic extract of *Scurrula ferruginea* still requires further investigation [86]. The investigations mentioned have utilized different inhibitors to delineate the plausible routes of mechanisms, but the molecular level details have not been elucidated.

A more detailed analysis of the methanolic extract of *Scurrula ferruginea* was further carried out by fractionating the methanolic extract fraction with ethyl acetate, chloroform and *n*-butanol. In vitro experiments showed that the *n*-butanol fraction of the methanolic extract of *Scurrula ferruginea* acts in a concentration-dependent mode to inhibit the isolated rat aortic rings’ contractions induced by phenylephrine (PE) and KCl. In vivo experiments with anesthetized adult male Sprague-Dawley rats showed that n-butanol methanolic extract of *Scurrula ferruginea* decreases the blood pressure in a dose-dependent mode for a more prolonged period. Chromatographic analyses of this fraction suggested the presence of terpenoid constituents [36]. Comparison of the different fractions of *Scurrula ferruginea* methanolic extract (ethyl acetate fraction, water fraction, chloroform fraction, n-butanol fraction) displayed that the n-butanol fraction of methanolic extract of *Scurrula ferruginea* has the highest EC_50_ value, the median concentration needed to bring about 50% reduction of the maximum contraction caused by the respective agonist, as reported from the concentration-response curve of methanolic extract of *Scurrula ferruginea*. The R_max_ (maximal relaxation) responses values was also the highest for *n*-butanol fraction.

Ameer and team reported that the mode of vasorelaxant effect of the methanolic extract of *Scurrula ferruginea* is via reversible noncompetitive antagonism [88]. The study design made use of three positive controls and one control (NE) and the interpretation was made based on the y-intercepts of the double-reciprocal curve, which could be attributable to variations in experimental data collections. Further investigations to support the claim that the mode of action of the methanolic extract of *Scurrula ferruginea* is via noncompetitive antagonism are needed. However, identification of the exact bioactive component in the *n*-butanol methanolic extract of *Scurrula ferruginea* is needed. In addition, as the bioactive components responsible for the vasorelaxing effect have been suspected to be terpenoids, comparison of the mode of action profile of the methanolic extract of *Scurrula ferruginea* with those of known terpenoids would have yield precious insights into the mode of action [88]. The mode of action of the *n*-butanol methanolic extract of *Scurrula ferruginea* was mediated via muscarinic receptor stimulation of the endothelium, as removal of the endothelium diminished the vasorelaxant effect exerted by the extract. The biological active component in the *n*-butanol methanolic extract of *Scurrula ferruginea* was found to activate the NO-cGMP pathway and/or enhancement of prostacyclin PGI2 release with possible cross-signaling modified by responses to Acetylcholine and sodium nitroprusside or stabilizing nitric oxide half-life. Chemical analysis of the *n*-butanol methanolic extract of *Scurrula ferruginea* reported 0.4% of flavonoids, 6.3% phenolic compounds, 0.3% total antioxidant activity and 0.04% free radical scavenging activity [36]. In summary, *Scurrula ferruginea* is a parasitic plant with medicinal effects in the treatment of antihypertension. Exploration of the compounds contained within this plant may unravel a wide array of therapeutics.

On the other hand, the antihypertensive effect of *Viscum album* was recorded in 1907 by a French physician, Renѐ Gaultier. Viscysate, a commercially available mistletoe preparation from around 1930s to 1950s, was used as a vasodilator [129]. Nevertheless, only in the recent decade, antihypertensive work on *Viscum album* re-emerged, resurrecting the evidence of vasodilation of the genus *Viscum*. In 2007, a study by Ofem et al. indicated that the leaves extract of *Viscum album* induced a significant decrease in blood pressure without causing any change in heart rate. This led the team to postulate that *Viscum album* most likely decrease blood pressure via the sympathetic pathway [130]. A pilot study on the assessing the antihypertensive effect of *Viscum album* also showed promise [131]. It is noted that the anticancer and antioxidative effects of *Viscum album* were mentioned and these activities are facilitated by the phytochemicals, such as lectins, phenylpropanoids and flavonoids in these mistletoes. Relatedly, these phytochemicals also promote lowering of blood pressure and extracts of *Viscum album*, especially aqueous extract, have been used in treatment of hypertension [132]. In addition, isolated oleanolic acid, a triterpenoid, from the cuticular wax of the hyperparasite *Viscum articulum* has been noted to exert antihypertensive effects in hypertensive rats by diuresis and nephroprotection [133].

Thus, both genera of mistletoes *Scurrula* and *Viscum* showed evidence of exerting vasodilation effects and are potential antihypertensive drug. However, further clinical trials to ensure the safe use and efficacy of these extracts are still necessary and yet to surface.

### 4.5. Weight Loss

Obesity, a metabolic disorder is getting increasingly prevalent globally, leading to various comorbidities and has been linked to mortalities as well [134]. Beneficial values of traditional herbal medicines for obesity have been well documented [135]. It has been established that inhibition of enzyme, fatty acid synthase (FAS), leads to reduction in body weight due to the lesser production of fatty acids in the body [136]. Both reversible and irreversible FAS inhibition were tested for sixteen different medicinal plants of Loranthaceae and Viscaceae families. Loranthaceae family had 400-fold greater activity by reversible inhibition of the enzyme. Furthermore, *Taxillus chinensis* exhibited the best activity on both mechanisms of FAS inhibition [78]. It was interesting to observe that different host plants did not affect the FAS inhibition. The study was continued with animal experimentation with oral administration of the plant extract which showed best activity, which yielded significant reduction of weight by decreasing food intake. While the control animal group gained about 5%, the treatment group lost about 9.8% relative to their initial body weight. FAS is linked to feeding regulation, and was shown to reduce neuropeptide Y, which promotes appetites. Thus it was hypothesised that decrease in fat production by FAS inhibition combined with appetite reduction would lead to balance shifting of energy production and would lead to reduction in body weight [78]. However, systematic scientific studies on the benefits described as indigenous usage for obesity of other plants needs to be further explored for the benefit of society.

### 4.6. Post-Partum Use

*Scurrula ferruginea* Danser has been mentioned to be used after delivery in various indigenous medicines [52,137]. However, scientific and medicinal evidence has not been found in the literatures for this use.

### 4.7. Gastrointestinal Effects

It is well known that herbal remedies are used worldwide for gastrointestinal disorders. Although there are modern medications available, the herbal remedies are widely used for their efficacy, ease of use and most importantly for lesser side effects. The flowers and leaves of *Scurrula ferruginea* has been reported for its therapeutic purpose, as a purgative while the haustorium were noted for their effect on ulcer [96].

A preliminary study of the *Scurrula ferruginea* methanolic extract on gastrointestinal effects exhibited dose-dependent spasmogenic activity [86]. Subsequently, to elucidate the mechanism of action of the contractile responses in isolated guinea pig ileum, graded additions of the extracts were analysed with different pharmacological interventions. The extracts inhibited the contraction by atropine while the opposite was true of neostigmine; additionally, it was unchanged by hexamethonium. Thus considering the abovementioned pharmacological results, it was postulated that the extract mimics acetylcholine, works directly on the ileal muscarinic receptors and acts as a substrate for the acetylcholinesterase enzyme. Acetylcholine is the major excitatory neurotransmitter regulating gut motility, it activates the M3 receptor subtype in smooth muscles [96]. Although the extract acts through activation of muscarinic receptor achieving cholinomimetic activity, it is still not evident whether the individual phytoconstituents would have similar effect.

### 4.8. Toxicity Studies

Lethal dose (LD_50_) is calculated as the dose required to kill 50% of a species. This data is essential to identify the optimal therapeutic dose and the highest dose up to which the extract can be given, above which lethality would be expected. The toxicity studies can be acute, sub-acute and chronic, where the former is the most commonly used type to evaluate the dose to be used for test dose for preliminary testing.

As observed by Mothana, various doses of *Loranthus regularis* methanolic extract did not present mortality or morbid symptoms up to 1500 mg/kg orally. The animals were orally administered with graded doses of 500, 1000 and 1500 mg/kg in their respective groups. Thus LD_50_ values could not be determined as there were no mortalities [89]. While another study confirmed the safety of *Loranthus micranthus* up to 5000 mg/kg [85]. Yet another study on the same plant leaves was administered orally up to and 827 mg/kg body weight and was observed for biochemical parameters, like alanine transaminase, aspartate transaminase, cholesterol, glucose, bilirubin, urea, protein and alkaline phosphatase. Neither adverse biochemical changes nor mortality was detected [138]. Brine shrimp (*Artemia salina*) lethality test, a bioassay, to assess in vitro toxicity was conducted on water and methanolic extracts of *D. pentandra* wherein the lethality was observed after a day of exposure and were found to be non-toxic [137].

A systematic review of 48 animal experiments reported about higher doses of *Viscum album* extracts or isolated mistletoe lectins, with the former parasite given at 1400 mg/kg. The isolated mistletoe lectins were administered subcutaneously, nasally and orally, at a dose of 14 μg/kg, 50 μg/kg and 500 μg/kg, respectively. In another study, immunostimulatory response was observed accompanied by substantial increase of cellular and humoral response after antigen stimulation. While there were five studies reporting lethality of animals after high dose in *Viscum album*, only one study reported death with mistletoe lectin in mice [139]. Therefore in the light of evidence about death at higher doses, it is important to study toxicity at the doses intended for therapeutic dose and should be thoroughly studied for allergic reactions as well.

## 5. Clinical Trials

Herbal medicines are consumed by cancer patients, both with and without prescription in German-speaking countries and was investigated by National Center for Complementary and Alternative Medicine (NCCAM) in 2003 [140].

### 5.1. Toxicity

An investigation through systematic review on the safety of administering extracts in humans was undertaken. There were 69 clinical studies with higher doses of *Viscum album* extracts, administered by different routes, and intravenous isolated mistletoe lectins, 1500 mg/kg and 6.4 μg/kg, respectively. For both, although immunosuppressant activity was not observed, occasional allergic reactions were reported, while for recombinant mistletoe lectins anaphylactic reactions and reversible hepatotoxicity were detected [139].

### 5.2. Immunological Studies

Fermented *Viscum album* extract is commercially available as Iscador^®^. While, Iscador is a registered homeopathic drug in United Kingdom, France and Italy, the extract preparation is approved in Austria, Germany, Sweden, Switzerland, and Georgia as an adjuvant for palliative cancer treatment by subcutaneous administration [141]. This was used in a six months observational study to evaluate the effect of swift and slow escalation dose regimes on T lymphocytes functionality in 71 patients with various carcinomas, namely breast, prostate and colorectal. A noticeable reduction was observed in prostate or colorectal cancer patients’ stimulated T-cell function. Interestingly, this was in contradiction to the results not only in breast cancer patients, who received lower average concentrations per month but also in patients whose doses were altered due to strong local reactions. It was concluded that Iscador^®^ should be based on patients’ personal adaptation characteristics following personal medicine principles rather than standard dose regime [142].

Mistletoe lectins are considered to be effective in improving the quality of life during chemotherapy or radiotherapy [143]. In order to test the local reactions and alternations in immune system, two of the popular preparations were tested. Iscucin^®^ Populi (IP) and *Viscum* *mali* e planta tota (VM), are the preparations from mistletoe, growing on poplar and apple tree, respectively. A 3-armed randomized, double blind clinical trial was conducted on healthy volunteers, who received increasing doses of either VM (1:1000, 1:100 and 2%) or IP (0.0125%, 0.25% and 5%) at 3 different doses for respective groups or placebo administered by subcutaneous route biweekly for 12 weeks. IP strengths higher than 0.1025% caused strong local reaction at injection site and eosinophilia. All IP concentrations increased CD4 counts without IL-6 and CRP increase. However, VM 2% produced mild local reaction and a small increase in eosinophil cell count [144].

### 5.3. Cancer

Non-randomized (16 studies), randomized (19 studies) controlled studies and single arm cohort studies fit the criteria for the systematic review on breast and gynaecological cancer treatment by *Viscum album* extracts, which were well tolerated. The extract and its’ compounds showed significant cytotoxic effect on in vitro cancer cells and in animal studies. Likewise, evidence of positive effects on both carcinomas was reported in the controlled studies when high dosage was given locally [145].

Despite a range of cytotoxic, radiation and surgical therapies available for breast cancer, breast cancer mortality has not reduced significantly. Thus complementary and alternative medicine (CAM) is being considered in various centres to improve QoL and reduce drug side effects [146]. Various studies examined the use of CAM to optimise the therapy, among which mistletoe extract was recommended as it had minimal side effects, although there was incidence of allergic reactions in few patients [147]. The evaluated trials demonstrated the possibility that a blend of motivational and pharmacological factors, especially cellular and humoral immune response enhancement were responsible for the positive clinical benefits with increased QoL observed in breast cancer patients due to the extracts [148].

A twelve month phase I/II clinical trial of aqueous mistletoe extract with 6 instillations per week to 30 superficial urothelial bladder cancer patients by intravesical administration followed by transurethral resection, cytology, cystoscopy, and random biopsies was conducted. Tumor-free patients were calculated to be 70%, while the rest had recurrence. There were no reports of side-effects, both local and systemic to extract standardized to mistletoe lectin, which showed good tolerability. Thus the study concluded that this extract is comparable to the standard adjuvant Bacillus Calmette-Guerin treatment, which has high side-effects, in effectiveness [149].

Abnoba*VISCUM*^®^ Q, a standardized mistletoe extract was evaluated for its safety and efficacy in 32 postsurgery patients with gastric carcinoma in a randomized controlled pilot study. The patients treated with doxifluridine were randomized either with or without the subcutaneous injection of test extract, triweekly from 1 to 24 weeks in increasing doses. Eosinophil and leukocyte count from lab tests as well global health status from questionnaire increased significantly in the intervention group compared to control, with lesser frequency of diarrhoea in the former [150].

Advance pancreatic carcinoma patients who cannot tolerate first-line treatment or choose to decline main stream treatment, supportive care is available for improvement of quality of life or as palliative therapy, as they weigh the low success probability against side effects of conventional treatment side effects [151]. In addition, a colon adenoma was reported to regress completely following intratumoural injection of *Viscum album* L. extract (Quercus; Iscador^®^ Qu) [74]. The case report, where an elderly Caucasian male who refused both adjuvant chemotherapy and surgery was administered twice with the extract, confirmed the complete disappearance of adenoma. Correspondingly, the biopsy revealed no adenoma or intraepithelial dysplasia [74].

Patients with pancreatic cancer either metastatic or locally advanced, who were only on supportive care and not on specific treatment were included in the randomised phase III trial for mistletoe extract, which was equivalent to the commercial product, Iscador Qu (*Viscum album* (L.) quercus). The dose escalation, given subcutaneously thrice a week, was continued until a local skin reaction accompanied with slight elevation of temperature was observed, the manifestations indicate optimal dose in the body. Patients treated with mistletoe extracts or with standard supportive care, were 96 and 72, respectively. The former group performed better not only in terms of pain, fatigue, loss of appetite and insomnia, with better confidence interval, but also in quality of life parameters [152].

The prospective, open label, randomised phase III, with late-stage pancreatic carcinoma patients for evaluating subcutaneous injection of *Viscum album* extract was undertaken by Tröger et al. The second arm of the trial was control patients who did not receive anticancer therapy. Dose escalation strategy was followed with thrice a week injection to the treatment arm while the both groups were given supportive care. Overall survival in the treatment group patients showed clinically relevant prolongation, which was well-tolerated and moreover exhibited less disease-related syndrome symptoms [153].

Management of malignant ascites is a significant challenge in advanced malignancies. Iscador M^®^ (*Viscum album* extract) instillation (10 mg) following paracentesis, to remove malignant peritoneal fluid, in peritoneal cavity of patients reduced the necessity of repeated paracentesis. A total of 23 patients with varying advanced stages, malignancies and histology were recruited for the Phase II study and were checked against self-baseline values for paracentesis requirement. Toxicity was not noticed and the time interval for paracentesis increased from 7 to 13 days, established the efficacy of plant extract in improving malignant ascites. Simple home care administration and feasibility to use in palliative end of life care were the additional advantages. A phase III randomised trial was recommended to validate the promising preliminary results [154].

### 5.4. Quality of Life

The research method quality of the controlled trials was assessed based on several criteria for herbal extract *Viscum album*. The influence of well-tolerated *Viscum album* extracts on the impact of quality of life (QoL) in cancer patients was evaluated in the systematic review. Quality of Life for the cancer patients was defined as, “how patients feel and function during the disease and its treatment, including psychosomatic self-regulation, subjective well-being, disease symptoms, performance status (Karnofsky Performance Status Scale), undesirable experiences associated with the use of chemotherapy or radiotherapy (adverse drug reactions), and “treatment symptoms”, by the reviewers of the controlled clinical studies [141]. The randomised controlled trials (RCTs) and non-RCT which fit the inclusion criteria of the reviewers were 26 and 10, respectively. The studies evaluated patient-reported QoL using well established and validated questionnaires. The studies were a mixture of either well designed or its’ contrary with major or minor methodological weakness. While non-RCTs reported a definitive QoL benefit, RCTs verdict was mixed. The areas benefitted by the *Viscum album* extract were exhaustion, nausea, appetite, anxiety and fatigue. Less consistent results were noticed for diarrhea, pain and general performance. It is beneficial to have controlled trials that are well designed and executed in order to reap benefits for the patients and for conclusive results [141].

## 6. Future Directions

There are many indigenous claims about these genera which have been used in traditional medicine. The claims if supported with scientific evidence backed by clinical trials would be beneficial for the community. Furthermore, in depth studies on pharmacological activity of the plant and its mechanism of action would be an additional advantage. Most of the suggested future studies have been mentioned under the appropriate sections. The plethora of compounds in these mistletoes means that these plants are a huge reservoir of potential medicinal compounds that can be used as leads to drug discovery. Further elucidation of parasitic plant response to host condition is needed. The knowledge of mechanism and biochemistry of each compound will minimise potential side-effects as well as maximize therapeutic potential of the drug. Despite widespread use of the genus for various medicinal purposes, evidence based pharmacological elucidation was conducted for specified extracts particularly for few activities. Moreover, the active phytoconstituent responsible for the biological activity needs to be identified and tested to further authenticate the claim. There is clearly a great need not only for patient but also for doctors to be educated about herbal remedies, which should be substantiated by randomized controlled trials for their safety and efficacy [155]. Moreover, there should be legislation for herbal quality control to ensure patient safety. Despite evidence from the preclinical studies about the significant effects on anti-tumour effects, there are reviews which suggest that there are very few clinical trials which are well-designed, thus questioning the reliability of the results of the trials. In general, adjuvant or supportive therapy from mistletoes is considered beneficial and safe improving the quality of life of solid tumours in adults. However, the evidence from reviews on mistletoe preparations as first line choice remains controversial. Therefore, there is an imminent need to substantiate whether the efficacy of mistletoes as evidenced in preclinical trials are transferred to a certain extend in human beings. It is recommended that systematic evaluations are to be conducted in prospective observational trials with well-designed randomised controlled trials with multidimensional quality of life questionnaires and focus group discussion as well as pharmacokinetic and pharmacodynamics comparisons of different preparations and dosages. For example prospective studies on *Viscum album* L. extract (Quercus; Iscador^®^ Qu) should evaluate to corroborate the reproducibility of the treatment effect and to investigate whether this approach could be a useful pre-operative intervention for colon adenomas which are too large for endoscopic resection. Phase III randomised trials would be the way to move forward for Iscador M^®^ which was found to be effective in the management of malignant ascites in phase II trials. It would be beneficial to the community to conduct systematic clinical trials.

## 7. Conclusions

Unlike *Viscum album* which has been intensively studied and translated into the drug Iscador, work on the mistletoes of Southeast Asia is still limited to ethnomedicinal uses. *Viscum album* extract has been studied in clinical trials and is being used in Europe as an adjuvant therapy for cancers of breast, malignant ascites and pancreas. Systematic literature review of 49 publications advocates that cancer patients treated with Iscador survives longer than non-treated patients [156]. Currently, there are no clinical trials for *Viscum album* targeted intratumoral delivery route as seen in clinicaltrials.gov. However, there was a recent article by Von Schoen-Angerer in 2014, which was a case report which described the complete regression of a colon adenoma following intratumoral injection of *Viscum album* [74]. As for *Scurrula* there are no known reports of intratumoral injection. The therapeutic effects of *Viscum album* infers a huge untapped potential of the *Scurrula* mistletoes due to the similarities of the plants from both genera. Plants of both genera have shown evidence of anticancer, antimicrobial, antioxidant and antihypertensive properties. As *Scurrula* research is still in its early stage, phytoconstituents isolation, structure activity relationship of phytoconstituents, mechanism of action of the bioactive compound and clinical trials are in the research pipeline. The abundance of phytoconstituents in plants of *Scurrula* genus evidently show potential for pharmacological drugs. Both *Scurrula oortiana* and *Viscum album* stimulate production of TNFα that assists the immune system to fight cancer. Also, the terpenoids in *Scurrula ferruginea*, which has been identified as the bioactive compound for its antihypertensive effect and is congruent to the finding as observed in triterpenoids of hyperparasite *Viscum articulatum*, Economically, Parasitic angiosperms can provide a valuable feedstock for pharmaceutical industry, therefore their medicinal values should be considered in taking account the nature of host species they are parasitizing as well as regional specificity. In conclusion, parasitic plants are important and useful, and future work to develop the *Scurrula* and *Viscum* genera mistletoes as reservoir of pharmacological drugs will prove worthwhile.

## Figures and Tables

**Table 1:** Parasitic mistletoe plants found in different host plants.

Parasite	Example(s) of Host Plant	Reference(s)
*Arceuthobium vaginatum* subsp. *cryptopodum*	*Pinus ponderosa*	[23]
*Dendrophthoe curvata* Blume	*Acacia auriculiformis* A. Cunn. Ex Benth *Andira inermis* (W. Wright) DC. *Mangifera indica* L. *Vitex pinnata* L.	[25,26]
*Phoradendron californicum*	*Acacia greggii*	[27]
*Phoradendron juniperinum*	*Juniperus osteospermum*	[27,28]
*Scurrula ferruginea*	*Tabebuia Rosea* *Tabebuia pallida*	[29]
*Viscum album*	*Acer platanoides* L. *Betula pendula Roth* *Salix alba* *Malus domestica*	[24,30]

**Table 2:** Vernacular Names of *Scurrula* genus.

Botanical Name	Synonym(s)	Common Name	Other Vernacular Name(s)	Reference(s)
*Scurrula oortiana*	*Dendrophthoe* *oortiana*(Korth.) Miq. *Loranthus* *oortianus* Korth.	Indonesian tea mistletoe	‘Benalu teh’	[31,32]
*Scurrula pulverulenta*	-	Powdery mistletoe	Leafy mistletoe	[33]
*Scurrula elata*	*Loranthus elatus* Edgew.	Butterfly-Bush mistletoe	Tall mistletoe	[34]
*Scurrula ferruginea*	*Loranthus ferrugineus* Jack	Rusty mistletoe	Akar naloe Benalu Dalu-dalu Dedalu api merah Dedalu api gajah Menalu asap Suridan	[9,35,36]
*Scurrula* *atropurpurea*	*Cichlanthus* *philippensis* (Cham. & Schltdl.) Tiegh. *Loranthus* *atropurpureus* Blume *Loranthus* *philippensis* Cham. & Schltdl. *Loranthus* *phillipensis* var. *macroantherus* Lecomte *Scurrula* *philippensis* (Cham. & Schltdl.) G. Don	Indonesian tea mistletoe	‘Benalu teh’	[37]

**Table 3:** Chemical structures of compounds found in *Scurrula* plants.

Name	Structure	Reference(s)
***Scurrula ferruginea***		
Quercetin	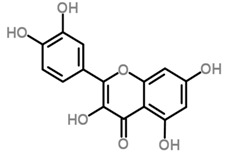	[41]
Quercitrin	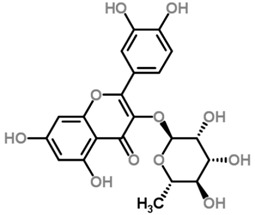	[41]
4-*O*-acetylquercitrin	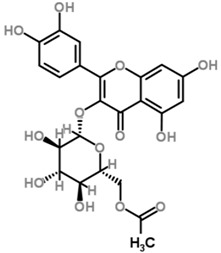	[41]
***Loranthus parasiticus***		
Coriamyrtin	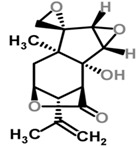	[45]
Tutin	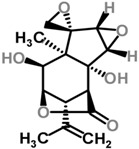	[45]
Corianin	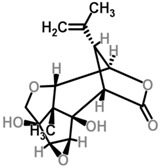	[45]
Coriatin	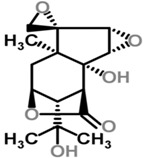	[45]
Proanthocyanidins of (+)-catechin	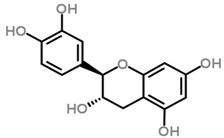	[45,46]
Proanthocyanidins of AC trimer	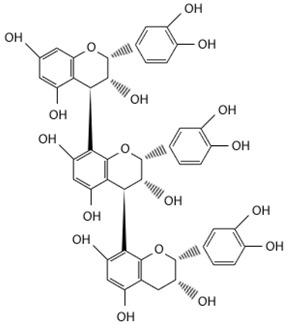	[45]
***Scurrula atropurpurea***		
*Z*-octadec-12-ene-8,10-diynoic acid	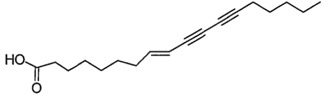	[46]
octadeca-12-ene-8,10-triynoic acid	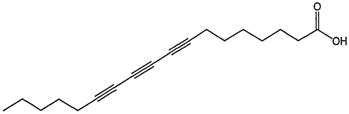	[47]
Flavanes-Epigallocatechin-3-*O*-gallate	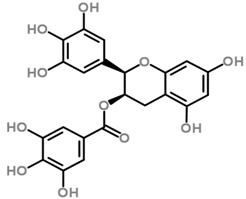	[46]
***Scurrula parasitica***		
Quercetin 3-*O*-β-l-galactopyranoside	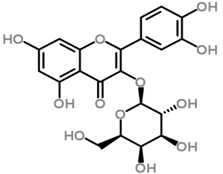	[48]
Quercetin 3-*O*-β-l-arabinopyranoside	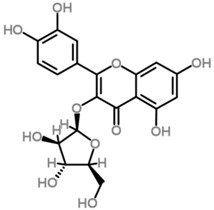	[48]
Quercetin 3-*O*-α-l-rhamnopyranoside	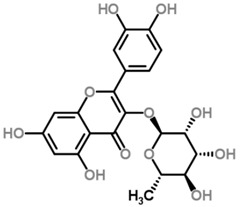	[48]

**Table 4:** Biological activities of compounds isolated from the parasitic plants.

*Plant*	*Parts of Plant Used*	*Type of Extract(s)*	*Observation(s)*	*Bioactive Compounds*	*Reference(s)*
***Antibacterial Activity***					
*Scurrula atropurpurea*	Leaves Stems	Ethanol extract	Antibacterial in inhibiting the growth of *Enterobacter sakazakii*	Flavonoids—inhibition bacterial cell wall Triterpenoid—inhibition protein synthesis Tannin	[81]
*Scurrula ferruginea*	Leaves Stems Flowers	Aqueous extract	Antibacterial activity against (MIC) Staphylococcus aureus Bacillus subtilis Escherichia coli Pseudomonas putida	Phenolic compounds	[82]
*Macrosolon cochichinensis*	Leaves Stems Junction to its hosts (Haustorium)	Methanolic extract and aqueous extract	Antibacterial activity (with inhibition zones from 4 mm to 8 mm) against Bacillus subtilis Klebsiella pneumonia Vibrio cholera	-	[83]
*Scurrula atropurpurea*	Leaves Stems Junction to its hosts (Haustorium)	Methanolic extract	Antibacterial activity against *Bacillus subtilis* (with inhibition zone of 5–6 mm) *Escherichia coli* (with inhibition zone of 4–5.5 mm) *Klebsiella pneumonia* (with inhibition zone of 6–7.5 mm) *Vibrio cholera* (with inhibition zone of 7–8 mm)	-	[83]
*Viscum album*	Leaves Stems Junction to its hosts (Haustorium)	Methanolic extract	Antibacterial activity against *Klebsiella pneumonia* with inhibition one of 3–3.5 mm	-	[83]
		Aqueous extract	Antibacterial activity against *Bacillus substilis* with inhibition one of 2–3 mm.	-	
*Loranthus micranthus*	Leaves	Crude methanol extract followed by fractionation with ethyl acetate and acetone	Antibacterial activity against Staphylococus aureus Bacillus subtilis Pseudomonas aeruginosa Escherichia coli	Alkaloids Flavonoids Terpenoids Tannins	[84]
***Anticancer or cytotoxic***					
*Scurrula ferruginea*	leaves stems twigs flowers	Extracted with petroleum Ether followed by isolation from ethyl acetate fraction	Quercetin was found to be the most active in the following four human cancer lines: U251 (NCI strain) K562 (CCL-243, ATCC) DU145 (HTB-81, ATCC) MCF-7 (HTB-22, ATCC)	Quercetin Quercitrin 4-*O*-acetylquercitrin	[41]
*Loranthus micranthus*	Leaves	Aqueous extract	Genotoxic effects against *Allium cepa* root cells	Alkaloids Flavonoids Tannins Phenolics Steroids Saponins Cardiacglycosides Reducing sugars	[85]
*Loranthus parasiticus*	-	The methanol extract, ethyl acetate and aqueous fractions	Cytotoxic against the ovarian cancer cell lines, namely SKOV3, CAOV3 and OVCAR-3	Proanthocyanidins of AC trimer Proanthocyanidins of (+)-catechin Coriamyrtin Tutin Corianin Coriatin	[45]
*Scurrula oortiana*	Leaves Stems	Aqueous and methanolic extracts	WEHI-164 cells sensitive to TNFα when treated with extract	-	[31]
*Scurrula atropurpurea*	Leaves Stems	Extracted with 70% acetone followed by fractionation with ethyl acetate	Octadeca-8,10,12-triynoic acid was most potent against mesothelial cells isolated from Donryu rats	Octadeca-8,10-diynoic acid *Z*-octadec-12-ene-8,10-diynoic acid Octadeca-12-ene-8,10-triynoic acid Epigallocatechin-3-*O*-gallate	[47]
*Scurrula atropurpurea*	-	Preparation of C_16_-Alkynic fatty acid	inhibitory effects on cancer cell invasion assay mesothelium monolayer by using MM1 cell line isolated from rat ascites hepatoma AH130 cells	Hexadeca-6,8,10-triynoic acid Hexadeca-8,10,12-triynoic acid	[46]
***Anti-hypertensive***					
*Scurrula ferruginea Scurrula ferruginea*	Whole aerial plant	Crude methanol extract, chloroform extract, ethyl acetate extract	In vivo experiment: Vasorelaxant by using Rat thoracic aorta	Polyphenolic and Flavonoids compounds	[35]
		Methanolic Extract	Guinea Pig Ileum: Hypotensive and Spasmogenic effects	Polyphenolic and Flavonoids compounds	[86]
		Methanolic extract and by *n*-butanol fraction	Rat thoracic aorta rings: vascular smooth muscle relaxation in vitro and a dose-dependent hypotensive action in vivo.	Terpenoids	[87]
		Methanolic extract	The vascular effects of three different concentrations of this extract by reversible noncompetitive antagonism of norepinephrine-induced vasoconstriction	Terpenoids	[88]
		Methanolic extract and then successively fractionated using chloroform, ethyl acetate and *n*-butanol	The *n*-butanol fraction of LFME (NBF-LFME) was studied using isolated rat thoracic aorta: relaxation by stimulating muscarinic receptors, activating the endothelium-derived nitric oxide-cGMP-relaxant pathway	Terpenoids	[36]
***Antioxidant***					
*Loranthus parasiticus*	Leaves Branches	Water/aqueous extract followed by methanolic extract, then ethyl acetate extract.	Antioxidant activity by 2,2-diphenyl-1-picrylhydrazyl (DPPH) free radical scavenging activity	Sesquiterpene lactones: Coriamyrtin Tutin Corianin Coriatin Proanthocyanidins AC trimer and (+)-catechin	[45]
*Scurrula ferruginea*	Stem (highest anti-oxidant activity) Leaves Flowers (lowest anti-oxidant activity)	Acetone extract (mostly stem)	Antioxidant capacity of extracts were evaluated using DPPH free radical scavenging assay	Phenolic compounds	[82]
*Loranthus regularis Steud. ex* *Sprague*	Leaves Flowers Twigs	Ethyl acetate fraction of a methanol extract	The antioxidant power of the extract, its fractions and isolated compounds was studied using DPPH scavenging and b-carotene/linoleic acid tests	Quercetin 3-*O*-β-l-galactopyranoside Quercetin 3-*O*-β-l-arabinopyranoside Quercetin 3-*O*-α-l-rhamnopyranoside	[89]
*Scurrula parasitica*	Leaves (mature and tender)	Methanolic extract of the matured leaves	DPPH free radical scavenging assay	Phenolic compounds	[90]
*Loranthus parasiticus*	Leaves	Ethanolic extract and further partitioned into ethyl acetate fraction and followed by aqueous fraction	Measurement of intracellular reactive oxygen species (ROS); H_2_O_2_-induced oxidative damage in NG108-15 cells	Proanthocyanidins AC trimer and (+)-catechin	[91,92]
*Viscum album*	Leaves Stem	Aqueous extract Ethanolic extract Acetone extract Methanolic extract (leaves rich in phenolics and carotenoids)	DPPH free radical scavenging assay ORAC method TEAC method Folin-Ciocalteu FRAP method DCFH-DA assay (to measure intracellular ROS levels)	Phenolic acids	[24,93,94]
***Antiviral***					
*Scurrula ferruginea*	Leaves Stems Flowers	Methanolic extracts	Antiviral activity against poliovirusactive on Poliovirus and activity on the U251 glioblastoma cells	Flavonoids Condensed Tannins	[52]
*Loranthus parasiticus*	Leaves	Methanolic extracts	Anti HIV-1 effect	-	[95]
*Scurrula oortiana*	Leaves Stems	Aqueous extracts	Anti Marek’s Disease Virus (MDV)	-	[32]
***Neuroprotection***					
*Loranthus parasiticus*	Leaves	Aqueous fraction	Neuroprotective role in NG108-15 cells	Proanthocyanidins of AC trimer	[91]
		Aqueous fraction	Increased cell viability and decreased intracellular ROS level in a dose-dependent manner against H_2_O_2_-induced oxidative stress in NG108-15 cells	Proanthocyanidins of (+)-catechin	
***Anti-schizophrenic Activity***					
*Loranthus parasiticus*	Leaves	Ethanol extract	Coriamyrtin with strong catatonic action in mice were effective components of *L. parasiticus* for shock therapy in catatonia treatment.	Coriamyrtin	[45]
			Non-toxic corianin with comparable activity to electric shock or insulin has been used for catatonia treatment by muscle injection in hospitals of various areas of China.	Corianin (non-toxic)	
			Tutin with strong catatonic action in mice were effective components of *L. parasiticus* for shock therapy in catatonia treatment.	Tutin	
***Miscellaneous studies***					
*Scurrula ferruginea*	Whole aerial plant	Methanolic Extract	Spasmogenic effect in isolated guinea pig ileum	Flavonoids Terpenoids Saponin Tannins	[86]
		*S. ferruginea* methanol extract and followed by ethyl acetate and *n*-butanol fraction	In vitro Cholinomimetic Effect		[96]
*Taxillus chinensis*	Stem	Extracted with 50% ethanol in the ratio 1:20 (*w/v*)	Potent inhibition on fatty acid synthase (FAS) that is proposed to be a potential therapeutic target for treatment of obesity.	-	[78]

Cell lines: 3LL—Lewis lung carcinoma cells, DU145—human prostate cancer cell line, K562—human leukemic cell line, L1210—mouse lymphocytic leukemia cells, MCF-7—human breast adenocarcinoma cell line, NG108-15 cells—neuroblastoma-glioma hybrid cells, U251—Human glioblastoma astrocytoma cell line.
